# CHANGING PATTERNS IN THE EPIDEMIOLOGY OF ACUTEGASTROINTESTINAL BLEEDING IN BRAZIL OVER THE LAST 12 YEARS

**DOI:** 10.1590/S0004-2803.24612025-064

**Published:** 2026-03-02

**Authors:** Gabriel Amorim Soares PEREIRA, Maria Luiza Barbosa FERREIRA DA SILVA, Amanda Albuquerque FARIAS DA SILVA, Fernanda Almeida da Silva de Sá OLIVEIRA, Danilo Costa Marques da Silva VASCONCELLOS, Fernanda Sales Melo MENDES, Liana CODES, Paulo Lisboa BITTENCOURT

**Affiliations:** 1 Bahiana School of Medicine and Public Health, Salvador, BA, Brazil.; 2 Portuguese Hospital, Unit of Gastroenterology and Hepatology, Salvador, BA, Brazil.

**Keywords:** Gastrointestinal bleeding, etiology, epidemiology, mortality, Brazil, Sangramento gastrointestinal, etiologia, epidemiologia, mortalidade, Brasil

## Abstract

**Background:**

Gastrointestinal bleeding (GIB) is one of the leading causes of hospitalization attributed to digestive disorders. Little is known about etiology and outcomes of GIB and temporal trends in the incidence of upper GIB (UGIB) and lower GIB (LGIB) in Brazil.

**Objective:**

To investigate the main causes and mortality of patients admitted to a tertiary care hospital in Brazil with UGIB and LGIB, as well as to assess trends in epidemiology and outcomes of GIB over time.

**Methods:**

All patients admitted to the Gastrointestinal (GI) Unit of the Portuguese Hospital of Salvador, Bahia, Brazil with the diagnosis of GIB between January 2012 and December 2023 were retrospectively investigated. All patients with GIB were classified as non-variceal (NUGIB), variceal (VUGIB) UGIB and LGIB according to standard criteria and managed according to an institutional protocol. Demographics, type and etiology of GIB and in-hospital mortality were evaluated in two different periods, between 2012-2017 (period 1) and 2018-2023 (period 2).

**Results:**

2.145 patients (1.214 males, mean age 70+16 years) were admitted, 1.185 in period 1 and 960 in period 2. Most of the patients had hematochezia and melena. NUGIB, VUGIB, LGIB and mid-GIB were observed in 37.5%, 14.4%, 40.3% and 5.6% of the patients, respectively. The remaining 47 subjects were not investigated due to advanced age or comorbidity. The most common etiologies for UGIB and LGIB were, respectively, esophagogastric varices (EV), duodenal (DU) and gastric ulcer (GU), and colonic diverticular disease (CDD), actinic proctocolitis (APC) and hemorrhoids (HE). Changes in the frequency of LGIB (42.1% vs 38.0% in period 2, *P*<0.0001) and mid-GIB (3.8% vs 7.9% in period 2, *P*<0.0001) were recorded over time. Age (68.7+15.6 vs 71+15.7 years in period 2, *P*=0.001) and gender (54,1% vs 59.1% of males in period 2, *P*=0.01) were also shown to vary as well as a significant decrease in mortality in recent years (14,2% vs 10.1% of deaths in period 2, *P*=0.005).

**Conclusions:**

EV, DU, GU and CDD, APC and HE were the most frequent causes of UGB and LGIB, respectively. Shifts in demographics, frequency of LGIB and mid-GIB and mortality were demonstrated in recent years.

## HIGHLIGHTS

• In this largest Brazilian cohort to date, esophagogastric varices, peptic ulcers, and colonic diverticular disease were the leading causes of gastrointestinal bleeding (GIB).

• Over time, there was an increase in the number of admissions for GIB in older patients and male subjects, while in-hospital mortality significantly declined.

• A decrease in lower GIB and an increase in mid-GIB frequency were observed across the study periods.

## RESUMO

**Contexto:**

Hemorragia digestiva (HD) é uma das principais causas de hospitalização atribuída ao trato gastrointestinal. Pouco se sabe sobre a frequência das etiologia e seus desfechos, assim como as tendências temporais na incidência de HD alta (HDA) e baixa (HDB) no Brasil.

**Objetivos:**

Investigar as principais causas e mortalidade de pacientes internados em um hospital terciário no Brasil com HDA e HDB, bem como avaliar as tendências epidemiológicas e os desfechos ao longo do tempo.

**Métodos:**

Todos os pacientes internados na Unidade de Gastro-Hepatologia (UGH) do Hospital Português de Salvador, Bahia, Brasil, com diagnóstico de HD entre janeiro de 2012 e dezembro de 2023 foram investigados retrospectivamente. Os pacientes foram classificados como HDA, HDB, HDA não varicosa (HDANV) e HDA varicosa (HDAV) de acordo com os critérios padrão e tratados de acordo com um protocolo institucional. Dados demográficos, tipo e etiologia da HD e mortalidade hospitalar foram avaliados em dois períodos diferentes, entre 2012-2017 (período 1) e 2018-2023 (período 2).

**Resultados:**

2.145 pacientes (1.214 homens, idade média 70+16 anos) foram admitidos, 1.185 no período 1 e 960 no período 2. A maioria dos pacientes tinha hematoquezia e melena. HDANV, HDAV, HDB e HD média foram observadas em 37,5%, 14,4%, 40,3% e 5,6% dos pacientes, respectivamente. Os 47 indivíduos restantes não foram investigados devido à idade avançada ou comorbidades. As etiologias mais comuns para HDA e HDB foram, respectivamente, varizes esofagogástricas (VE), úlcera duodenal (UD) e gástrica (UG), e doença diverticular colônica (DDC), proctocolite actínica (PAC) e hemorróidas (HE). Mudanças na frequência de HDB (42,1% vs 38,0% no período 2, *P*<0,0001) e HD média (3,8% vs 7,9% no período 2, *P*<0,0001) foram registradas ao longo do tempo. Idade (68,7+15,6 vs 71+15,7 anos no período 2, *P*=0,001) e sexo (54,1% vs 59,1% dos homens no período 2, *P*=0,01) também apresentaram variações, bem como uma redução significativa na mortalidade nos últimos anos (14,2% vs 10,1% dos óbitos no período 2, *P*=0,005).

**Conclusões:**

EV, UD, UG e DDC, PAC e HE foram as causas mais frequentes de HDA e HDB, respectivamente. Mudanças na demografia, frequência de HDB e HD média e mortalidade foram demonstradas nos últimos anos.

## INTRODUCTION

Gastrointestinal bleeding (GIB) is a medical emergency often requiring hospitalization and admission in the intensive care unit (ICU), ranking among the top 10 causes of hospitalization attributed to digestive diseases^1^ ^-^ ^3^. It has been classified as upper (UGIB) or lower (LGIB), whenever the source of GIB was located proximal or distal to the ligament of Treitz, respectively^4^. More recently, LGIB was redefined as hemorrhage arising from the colorectum or anus, and the term mid-GIB has been adopted to encompass bleeding occurring in the small bowel between the ampulla of Vater and the ileocecal valve^5^. Upper GIB has been further divided into variceal (VUGIB) UGIB also known as portal-hypertensive hemorrhage, and non-variceal (NUGIB) UGIB, since both have separate management strategies and treatment options and different outcomes^6^ ^,^ ^7^.

The main causes of UGIB and LGIB were shown to vary according to the population studied. Peptic either duodenal (DU) or gastric ulcers (GU), erosive esophagitis (EE), gastric or duodenal erosions, esophagogastric varices (EV) and Mallory Weiss tears (MVT) are the most frequent lesions encountered at endoscopy in patients with UGIB, whereas colonic diverticular disease (CDD), hemorrhoids, colonic angiodysplasia, colorectal polyps and cancer and colitis are the most prevalent etiologies for LGIB worldwide^8^.

Incidence rates of UGIB and LGIB have been shown to range, respectively, from 15 to 172/100000 and 20.5 to 87/100000 person-years in different populations^9^. Several studies have reported a trend in UGIB, either NVUGIB or VUGIB, rate reduction in recent years driven by eradication of helicobacter pylori, broader access to proton pump inhibitor (PPI) therapy and therapeutic endoscopy, as well as prevention of portal hypertensive bleeding with the use of beta-blockers^2^ ^,^ ^3^ ^,^ ^8^ ^-^ ^12^. On the contrary, the incidence of LGIB has been reported to be increasing worldwide^8^ ^,^ ^9^ ^,^ ^12^, possibly due to the rising frequency of colonic angiodysplasia, colorectal polyps and cancer^8^. Mid-GIB due to small bowel angiodysplasia or tumors has been increasingly recognized after the advent and broader availability of capsule endoscopy and enteroscopy^13^.

Mortality rates were shown to vary widely in different populations^9^, but worse outcomes have been reported in older subjects, in patients with severe comorbidities or massive bleeding and in those with VUGIB, when compared to their counterparts with NUGIB^1^ ^,^ ^5^ ^,^ ^8^.

Few studies^14^ ^,^ ^15^ have evaluated the etiologies and outcomes of GIB in Brazil and to our knowledge none have investigated trends in the incidence of GIB over time. 

The purpose of the present study was to investigate the main causes and mortality of patients admitted to a tertiary care hospital in Brazil with UGIB and LGIB, as well as to assess trends in epidemiology and outcomes of GIB over time. 

## METHODS

All patients admitted to the Gastroenterology and Hepatology Unit of the Portuguese Hospital of Salvador, Bahia, Brazil with the diagnosis of GIB between January 2012 and December 2023 were retrospectively investigated. This intensive care unit is a referral center for privately insured patients with GIB in the metropolitan area of Salvador, Bahia. 

According to an institutional protocol, all patients admitted to the emergency room with hematemesis, melena or hematochezia are granted access to this ICU for fluid resuscitation, pharmacological therapy and appropriate interventions including endoscopy, interventional radiology and surgery whenever required. All patients were managed according to our GIB guidance, published elsewhere^16^ ^-^ ^18^, which has been constantly updated according to national and international guidelines^6^ ^,^ ^7^ ^,^ ^19^.

Upper gastrointestinal endoscopy and/or colonoscopy were performed in all eligible patients based on clinical judgement for etiology assessment and endoscopic treatment whenever indicated. Patients who refused to endoscopy and/or colonoscopy or were ineligible due to advanced age or comorbidity were not investigated. All GIB episodes were classified as NUGIB, VUGIB and LGIB according to predefined criteria. Subjects with portal-hypertensive bleeding, irrespective of the presence of varices, were considered to have VUGIB. Mid-GIB was considered arbitrarily in the absence of a source of bleeding in at least one endoscopy and one colonoscopy procedures. Subjects with melena or hematochezia unwilling to perform colonoscopy after a non-diagnostic upper gastrointestinal endoscopy were considered as not investigated. Few patients underwent either capsule endoscopy or enteroscopy for further diagnostic assessment since both procedures were not covered during the study period by most private health insurances in Brazil.

Data regarding demographics, type and etiology of GIB and in-hospital mortality were recorded. Patients were followed until death or hospital discharge. The primary outcome was in-hospital mortality. Mortality and etiology of GIB were additionally evaluated in two different periods, between 2012-2017 e 2018-2023.

The study adhered to the Declaration of Helsinki and was approved by the Ethics Committee for Research at Hospital Português in Salvador, Bahia.

### Statistical analysis

Dichotomous variables are presented in text and tables as numbers and percentage and continuous variables were expressed as mean ± standard deviation (SD) or as median and interquartile range, respectively, whether the distribution was normal or skewed. Demographic, clinical, and laboratory variables were compared using the chi-square test or Fisher’s test, whenever appropriate, for categorical variables or Student’s t-test or the Mann-Whitney U test for continuous variables when appropriate. A *P*-value <0.05 was considered significant. Statistical analyses were performed with the Statistical package for social sciences (SPSS Inc., Chicago, IL, USA), version 21.0 for Windows.

## RESULTS

Two thousand one hundred forty-five patients (1.214 males, mean age 70+16 years) were admitted to the GI unit with GI bleeding between January 2012 and December 2023. One thousand one hundred eighty-five (55,2%) occurred in period 1, whereas 960 (44,8%) were observed in period 2. One thousand six hundred ninety-five (79,0%) patients were admitted once, whereas 450 (21%) had two or more admissions over time. Only 89 (4,1%) subjects had more than five admissions due to GI bleeding.

**FIGURE 1:**
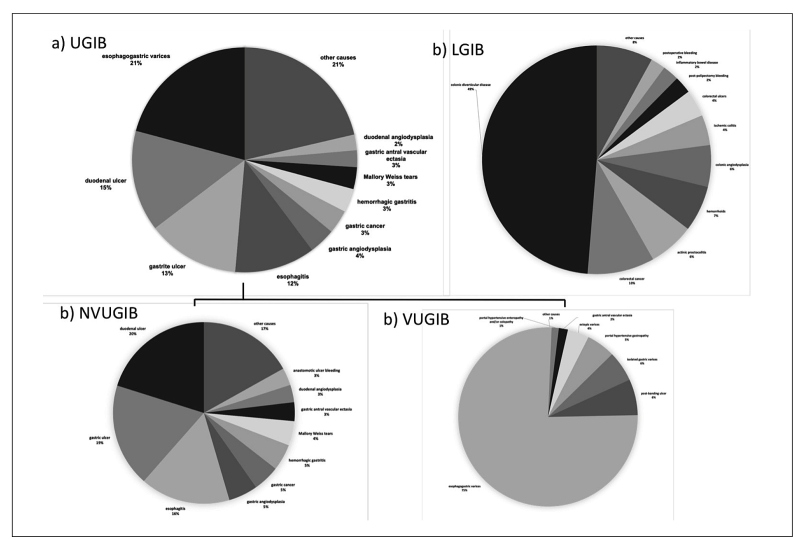
Main causes of a) UGIB including b) NVUGIB c) VUGIB and d) LGIB. UGIB: upper gastrointestinal bleeding. NVUGIB: non-variceal UGIB. VUGIB: variceral UGIB. LGIB: lower gastrointestinal bleeding.

Demographics, clinical features, and outcomes of those patients are outlined in Table 1. Briefly, hematochezia, melena, hematemesis, and coffee grounds vomiting were reported, respectively, by 848 (39,5%), 755 (35,2%), 402 (18,7%) and 140 (6,5%) of them. After upper and/or lower GI endoscopy, 1.113 (51,9%) GI bleeding episodes were characterized as upper GI bleeding, 805 (37,5%) as NUGIB and 308 (14,4%) as VUGIB. 864 (40,3%) patients had LGIB and 121 (5,6%) mid-GI bleeding. Forty-seven patients (2,2%) were not investigated due to comorbidity or advanced age at attending physicians’ discretion (TABLE 1). The top 10 causes of upper GI bleeding, including NVUGIB and VUGIB, and LGIB are depicted in Figure 1. Briefly, the most common etiologies for upper GI bleeding in general were EV, DU and GU, EE, gastric angiodysplasia, hemorrhagic gastritis, gastric cancer, MVT, gastric antral vascular ectasia and duodenal angiodysplasia (Figure 1). After stratification of upper GI bleeding into NVUGIB and VUGIB by endoscopy, leading causes of NUGIB and VGIB, also encompassing other causes of portal hypertensive bleeding beyond varices, were, respectively, DU and GU, EE, gastric angiodysplasia, hemorrhagic gastritis, gastric cancer, MVT, gastric antral vascular ectasia, duodenal angiodysplasia and anastomotic ulcers (Figure 1).

**TABLE 1:** Demographics, clinical features and outcomes of patients hospitalized with gastrointestinal bleeding.

	All patients (n=2.145)
Demographics	
Age	70+16
Male Sex	1.214 (56.6%)
Symptoms	n (%)
Hematochezia	848 (39.5%)
Melena	755 (35.2%)
Hematemesis	402 (18.7%)
Coffee grounds vomiting	140 (6.5%)
Source of bleeding	n (%)
UGIB	1.113 (51.9%)
NUGIB	805 (37.5%)
VUGIB	308 (14.4%)
LGIB	864 (40.3%)
Mid-GIB	121 (5.6%)
Not investigated	47 (2.2%)
Hospital LOS (days)	3 [1-5]
Mortality	265 (12.4%)

GIB: gastrointestinal bleeding. UGIB: upper GIB. NUGIB: non-variceal UGIB. VUGIB: variceal UGIB. LGIB: lower GIB. LOS: length of stay.

In respect to LGIB, CDD, colorectal cancer, actinic proctocolitis (APC), hemorrhoids, colonic angiodysplasia, ischemic colitis, colorectal ulcers, post-polypectomy bleeding, inflammatory bowel disease and anastomotic ulcers were the most frequent lesions encountered (Figure 1).

The median APACHE score at admission was 11 [8-15] and the median hospital length of stay (LOS) was 3 [1-5] days. 265 (12,4%) patients died and 1880 were discharged alive. As expected, mortality was higher in those subjects with VUGIB (19,1%) when compared to their counterparts with NUGIB (14,6%), LGIB (7,2%) and Mid-GIB (5,9%) (*P*<0,0001).

**FIGURE 2:**
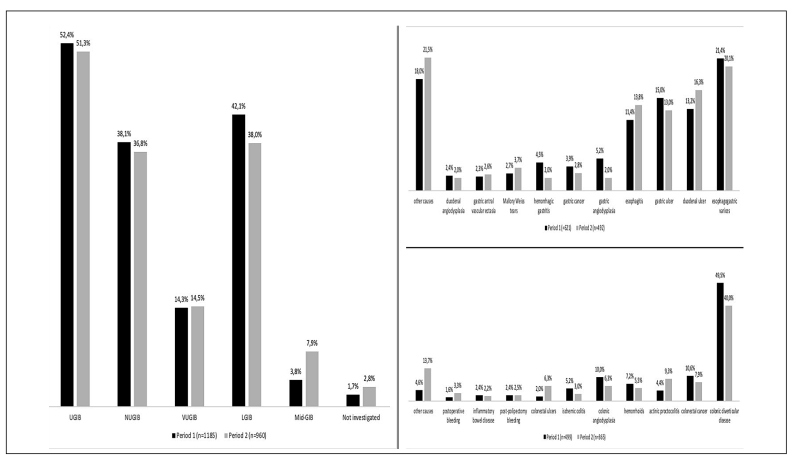
Frequency and leading causes of GIB over time.

Comparison of demographics, clinical features, and outcomes according to the admission period revealed that patients admitted in period 2 were significantly older and more frequently males. In addition, they stayed longer in the hospital and had lower mortality, when compared to their counterparts in period 1 (Table 2). The distribution of GIB types over time remained stable for UGIB, NUGIB and VUGIB, while the frequency of LGIB declined and Mid-GIB increased in period 2, when compared to period 1 (Figure 2). The distribution of the top 10 etiologies for UGIB also remained relatively stable over time, whereas the frequencies of CDD decreased and APC increased over time as causes of LGIB. 

**TABLE 2:** Demographics. clinical features and outcomes of patients hospitalized with gastrointestinal bleeding over time.

	Period 1 (n=1185)	Period 2 (n=960)	*P*
Demographics			
Age	68.7+15.6	71+15.7	0.001
Male Sex	641 (54.1%)	573 (59.7%)	0.01
Symptoms	n (%)	n (%)	0.23
Hematochezia	472 (39.8%)	376 (39.2%)	
Melena	433 (36.5%)	322 (33.5%)	
Hematemesis	206 (17.4%)	196 (20.4%)	
Coffee grounds vomiting	74 (6.2%)	66 (6.9%)	
Hospital LOS (days)	2 [1-66]	3 [1-51]	<0.0001
Mortality	168 (14.2%)	97 (10.1%)	0.005

LOS: length of stay.

## DISCUSSION

This study is the largest published cohort thus far of Brazilian patients admitted to the ICU with GIB. Most of the patients were males with advanced age with either hematochezia or melena at presentation. The top 5 etiologies for UGIB and LGIB were, respectively, EV, DU, GU and EE, and CDD, colorectal cancer, APC, hemorrhoids and colonic angiodysplasia. Overall mortality was 12,4%. As expected, death rates were higher in those subjects with VUGIB and NUGIB when compared to LGIB and Mid-GIB. To our knowledge, only two previous studies have investigated the etiology and outcomes of GIB in Brazilian patients, restricted to subjects with UGIB. Zalcman et al.^14^ have retrospectively investigated 324 consecutive admissions of patients with UGIB. Similar results were obtained concerning demographics and main etiologies of bleeding. Mortality was higher, particularly in those patients with liver disease. Maluf-Filho et al.^15^ reported another cohort of patients with UGIB and cancer. Dismal survival of those patients was attributed to advanced malignancy.

When compared to our results, mortality in the literature was shown to be lower, ranging from 0,7% to 4,8% and 0,5 to 8%, respectively, for UGIB and LGIB^9^. Those discrepancies may be due to overrepresentation in our cohort of patients of risk factors previously associated with worse outcomes, such as portal-hypertensive bleeding and advanced with significant comorbidity^21^ ^,^ ^28^ ^,^ ^29^.

The geoepidemiology of GIB was shown to vary in different parts of the world^20^ ^-^ ^27^. Overall, the most common causes of UGIB reported in medical literature were, respectively, peptic ulcer disease, gastritis or duodenitis, EE, EV, gastric or duodenal angiodysplasia and MVT, which were reported in 40-63%, 18-22%, 8-20%, 4-16% and 4-6% of the patients in several studies^20^, but differences in the hierarchy of main causes of UGIB were also disclosed in other studies, particularly in the frequency of MVT and bleeding EV^21^ ^,^ ^22^. In the present study, EV was the second most frequent bleeding lesion encountered after peptic ulcer. This may be due to differences in demographics, regional prevalence of underlying diseases, either cirrhosis or schistosomiasis, or local referral patterns. Likewise, the most frequent causes of LGIB in different reports from the UK and USA were CDD, hemorrhoids, colonic polyps and colitis, which were reported in 26-33%, 10-20%, 3-13% and 11-13% of the patients, respectively^23^ ^,^ ^24^. Variations in the distribution of main etiologies for LGIB were also reported in studies from other countries^25^ ^-^ ^27^, but the predominance of CDD, colorectal cancer and APC as main causes of LGIB in our study could be ascribed to the advanced age of the patients included in the present study.

As previously highlighted by others^9^ ^,^ ^21^ ^,^ ^23^, comparison of demographics, etiology, and outcomes of the patients with GIB admitted between 2012 to 2017 and 2018 to 2023 revealed differences in the epidemiology of GIB and clinical outcomes. Patients admitted more recently with GI hemorrhage were significantly older and more commonly males. Hospital length of stay was longer in the last period of observation probably due to the increasing age of the patients. The coronavirus disease pandemic may have also influenced the prolonged LOS of those subjects because many of them were discharged home directed from our ICU, which remained a COVID-free environment for admission of patients for elective and urgent surgery, decompensated cirrhosis and GIB between January 2020 and May 2023. Mortality, on the contrary, was significantly lower in the last period. Several evidence-based strategies, particularly more widespread use of through-the-scope clips for therapeutic endoscopy for NUGIB and early placement of transjugular intrahepatic portosystemic shunts for VUGIB, which were implemented in the last period may have contributed for better outcomes^6^ ^,^ ^7^ ^,^ ^16^ ^-^ ^18^.

In the present study, no change in the distribution of UGIB, either NUGIB or VUGIB, a decrease in the frequency of LGIB and an increase in the number of cases of mid-GIB was noted over time. Different trends were reported elsewhere^9^ ^,^ ^12^ ^,^ ^20^ ^,^ ^21^ ^,^ ^23^ ^,^ ^26^. Notably, several reports from various parts of the world reported a decrease in the incidence of UGIB, ascribed to widespread access to endoscopy, PPI therapy for peptic ulcer disease and helicobacter pylori eradication policies^9^. Data concerning trends in the incidence of LGIB remain much more inconsistent^9^. One recent study from the US^30^, using the nationwide emergency department sample, disclosed marked variations in incidence of GIB over time. The authors demonstrated an early decrease in the incidence of UGIB from 2006 to 2014 followed by a late increase thereafter until 2019^30^. Fluctuations over time were also reported in LGIB incidence, which may explain the discrepancies observed in other studies^8^ ^,^ ^9^.

Forty-seven out of 2.145 patients were not investigated due to advanced age or comorbidities. The decision to overlook a proper endoscopic diagnosis in those patients was based on careful bedside risk-benefit assessment. It is worth to mention that etiology could not be elicited in those subjects, but their number was too small to alter sample representativeness and the generalizability of the findings.

Mid-GIB is frequently caused by small bowel angiodysplasia or tumors, increasingly recognized after the advent and broader availability of capsule endoscopy and enteroscopy. The increase in the number of admissions for mid-GIB observed in the present study may therefore reflect a lack of access to diagnosis of mid-GIB associated lesions, since capsule endoscopy has been covered by private health insurance companies in Brazil only in recent years. Future studies incorporating these diagnostic modalities could provide a more accurate assessment of bleeding origin, particularly in cases of obscure or persistent hemorrhage.

In contrast to other reports^8^ ^,^ ^9^ ^,^ ^12^, no decrease in the frequency over time of peptic ulcer disease was disclosed in the present study. Different from other studies, which noted an increase in the frequency of CDD, colonic angiodysplasia, colorectal polyps and cancer in recent years, a reduction in number of admissions for CDD and an increase in the frequency of APC over time were observed in the present study. Those conflicting results may reflect distinct referral policies and admission patterns of those tertiary care centers taking care of patients with GIB.

The main limitation of this study is its retrospective design, which could lead to biases related to data collection and documentation. Retrospective analyses rely heavily on the accuracy and completeness of existing medical records, which may be subject to inconsistencies, missing data, or variations in how information was recorded across different healthcare professionals and time points. These factors should be considered when interpreting the results, although the real-world context and the size of the patient cohort contribute to the relevance of the observed patterns.

Another limitation of this study was the fact that it was conducted in a single tertiary hospital with a population mainly covered by private health insurance, which may limit the generalizability of the findings. Differences in patient profiles and healthcare access in public settings may lead to different outcomes. Therefore, caution is advised when applying these results to other populations. Lastly, another drawback of this study is the lack of adjustment for potential confounding variables, such as comorbidities and bleeding severity. These factors are known to influence clinical outcomes and could have affected our results. The absence of multivariate analysis limits the ability to identify independent associations. Further studies taking into consideration possible confounding variables in diverse healthcare settings are warranted.

In summary, in the largest cohort of Brazilian patients with GIB reported thus far, EV, DU, GU and CDD, APC and HE were the most frequent causes of UGB and LGIB, respectively. Temporal trends were observed in the frequency and etiology of LGIB. GIB is affecting older and male patients in recent years. Notwithstanding with that, mortality decreased significantly over the years, probably due to the widespread adoption in Brazil of evidence-based strategies, known to improve patient outcomes. 

## Data Availability

Data-available-upon-request
